# Surveillance of antibiotic resistance in Streptococcus spp in China-CHINET project 2007 and 2009

**DOI:** 10.1186/1753-6561-5-S6-P145

**Published:** 2011-06-29

**Authors:** W Chuanqing, CHINET project

**Affiliations:** 1 Nosocomial Infection Control, Children's Hospital of Fudan University, Shanghai, China

## Introduction / objectives

CHINET program, which started at 2005, is monitors bacterial antibiotic resistance in 12 China medical centers in 2007 and 14 China medical centers in 2009

## Methods

The susceptibility testing was carried out by unified protocol of Kirby-Bauer method (KB) were Streptococcus pneumonia (1699), β-hemolytic streptococci(1428) including GAS (756), GBS (451), GCS (34), GGS (140), and GFS (31) none classified (16), and Viridans streptococc group excluded S. pneumonia isolated from sterile parts (280). The susceptibility testing was assayed by Penicillin E-test were S. pneumonia and Viridans streptococcus. Results were analyzed according to CLSI2007 and 2009 criteria

## Results

Penicillin non-susceptible strains (PISP+PRSP) isolated from no bacterial meningitis patients in children aged < 5 year old group was 24.9%, and Erythromycin resistance was 96.9%, which were higher than that in ≥5 year old group (16.3%, 87.8% ) separately . Erythromycin and Penicillin resistance were 88.7%, 0% in GAS, 52.3% and 2.6% in GBS, 61.8% and 6.7% in GCS, 58.1% and 0% in GFS, 57.0% and 0.7% in GGS, 66.7% and 21.3% in Viridans Streptococci group. All isolates were highly sensitivity to Levofloxacin, Vancomycin, Linezolid, Moxifloxacin and Meropenem

## Conclusion

In conclusion, the resistant of S. pneumonia to penicillin is different between different age group. The resistant rates of streptococcus spp to erythromycin remain high in mainland China.

## Disclosure of interest

None declared.

